# New Books

**Published:** 2006-04

Advances in the Geological Storage of Carbon Dioxide

S. Lombardi, L.K. Altunina, S.E. Beaubien, eds.

New York:Springer, 2006. 362 pp. ISBN: 1-4020-4470-4, $79.95

Carbon and Its Domestication

A.M. Mannion

New York:Springer, 2006. 319 pp. ISBN: 1-4020-3956-5, $119

Chemical-Specific Adjustment Factors for Interspecies Differences and Human Variability

IPCS Harmonization Project Document No.2

Geneva:WHO Press, 2005. 100 pp. ISBN: 92-4-154678-6, $36

Comparative Think Tanks, Politics And Public Policy

James G. McGann, Erik C. Johnson

Northampton, MA:Edward Elgar Publishing, 2006. 304 pp. ISBN: 13-978-1-84376, $99

Ecotoxicology, Ecological Risk Assessment and Multiple Stressors

Gerassimos Arapis, Nadezhda Goncharova, Philippe Baveye, eds.

New York:Springer, 2006. 382 pp. ISBN: 1-4020-4474-7, $149

Environmental Health Impacts of Transport and Mobility

P. Nicolopoulou-Stamati, L. Hens, C.V. Howard, Eds.

New York:Springer, 2005,. 324 pp. ISBN: 1-4020-4304-X, $129

Environmental Sustainability: A Consumption Approach

Raghbendra Jha, K.V. Bhanu Murthy

Oxford, UK:Routledge, 2006. 208 pp. ISBN: 0-415-36346-2, $135

Free-Radical-Induced DNA Damage and Its Repair: A Chemical Perspective

Clemens von Sonntag

New York:Springer, 2006. 510 pp. ISBN: 3-540-26120-6, $299

From Cells to Proteins: Imaging Nature across Dimensions

V. Evangelista, L. Barsanti, V. Passarelli, P. Gualtieri, eds.

New York:Springer, 2005. 479 pp. ISBN: 1-4020-3614-0, $219

Fundamentals of Ecogenetics

Lucio G. Costa, David L. Eaton, eds.

Hoboken, NJ:John Wiley & Sons, 2006. 557 pp. ISBN: 0-471-46781-2, $110

Governing Environmental Flows: Global Challenges to Social Theory

Gert Spaargaren, Arthur P. J. Mol , Frederick H. Buttel, eds.

Cambridge, MA:MIT Press, 2006. 392 pp. ISBN: 0-262-19545-3, $67

Introduction to Sustainability: Road to a Better Future

Nolberto Munier

New York:Springer, 2005. 444 pp. ISBN: 1-4020-3557-8, $79.95

Microorganisms and Bioterrorism

Herman Friedman, Burt Anderson, Mauro Bendinelli, eds.

New York:Springer, 2006. 240 pp. ISBN: 0-387-28156-8, $129

Molecular Biology of Metal Homeostasis and Detoxification: From Microbes to Man

Markus J Tamás, Enrico Martinoia, eds.

New York:Springer, 2006. 509 pp. ISBN: 3-540-22175-1, $299

Protein Biochemistry and Proteomics

Hubert Rehm

Burlington, MA:Elsevier, 2006. 256 pp. ISBN: 0-12-088545-X, $44.95

Renewable Energies for Central Asia Countries: Economic, Environmental and Social Impacts

Aldo Iacomelli, ed.

New York:Springer, 2005. 182 pp. ISBN: 1-4020-3924-7, $119

Reviews of Environmental Contamination and Toxicology

George Ware, ed.

New York:Springer, 2006. 185 pp. ISBN: 0-387-25526-5, $109

Rising Above The Gathering Storm: Energizing and Employing America for a Brighter Economic Future

Committee on Prospering in the Global Economy of the 21st Century

Washington, DC:National Academies Press, 2006. 504 pp. ISBN: 0-309-10045-3, $70

The Landscape of Reform: Civic Pragmatism and Environmental Thought in America

Ben A. Minteer

Cambridge, MA:MIT Press, 2006. 272 pp. ISBN: 0-262-13461-6, $28

The p53 Tumor Suppressor Pathway and Cancer

Gerard P. Zambetti, ed.

New York:Springer, 2005. 246 pp. ISBN: 0-387-24135-3, $139

